# Characteristics and Application of *Rhodopseudomonas palustris* as a Microbial Cell Factory

**DOI:** 10.3389/fbioe.2022.897003

**Published:** 2022-05-12

**Authors:** Meijie Li, Peng Ning, Yi Sun, Jie Luo, Jianming Yang

**Affiliations:** ^1^ Energy-Rich Compound Production by Photosynthetic Carbon Fixation Research Center, Shandong Key Lab of Applied Mycology, Qingdao Agricultural University, Qingdao, China; ^2^ College of Life Sciences, Qingdao Agricultural University, Qingdao, China; ^3^ Haiyang Comprehensive Administrative Law Enforcement Bureau (Agriculture), Haiyang, China; ^4^ Qingdao Garden Forestry Technology School, Qingdao, China

**Keywords:** *Rhodopseudomonas palustris*, biopolymer, biofuel, wastewater treatment, microbial cell factory, photoautotrophic

## Abstract

*Rhodopseudomonas palustris*, a purple nonsulfur bacterium, is a bacterium with the properties of extraordinary metabolic versatility, carbon source diversity and metabolite diversity. Due to its biodetoxification and biodegradation properties, *R. palustris* has been traditionally applied in wastewater treatment and bioremediation. *R. palustris* is rich in various metabolites, contributing to its application in agriculture, aquaculture and livestock breeding as additives. In recent years, *R. palustris* has been engineered as a microbial cell factory to produce valuable chemicals, especially photofermentation of hydrogen. The outstanding property of *R. palustris* as a microbial cell factory is its ability to use a diversity of carbon sources. *R. palustris* is capable of CO_2_ fixation, contributing to photoautotrophic conversion of CO_2_ into valuable chemicals. *R. palustris* can assimilate short-chain organic acids and crude glycerol from industrial and agricultural wastewater. Lignocellulosic biomass hydrolysates can also be degraded by *R. palustris*. Utilization of these feedstocks can reduce the industry cost and is beneficial for environment. Applications of *R. palustris* for biopolymers and their building blocks production, and biofuels production are discussed. Afterward, some novel applications in microbial fuel cells, microbial electrosynthesis and photocatalytic synthesis are summarized. The challenges of the application of *R. palustris* are analyzed, and possible solutions are suggested.

## 1 Introduction

**FIGURE 1:**
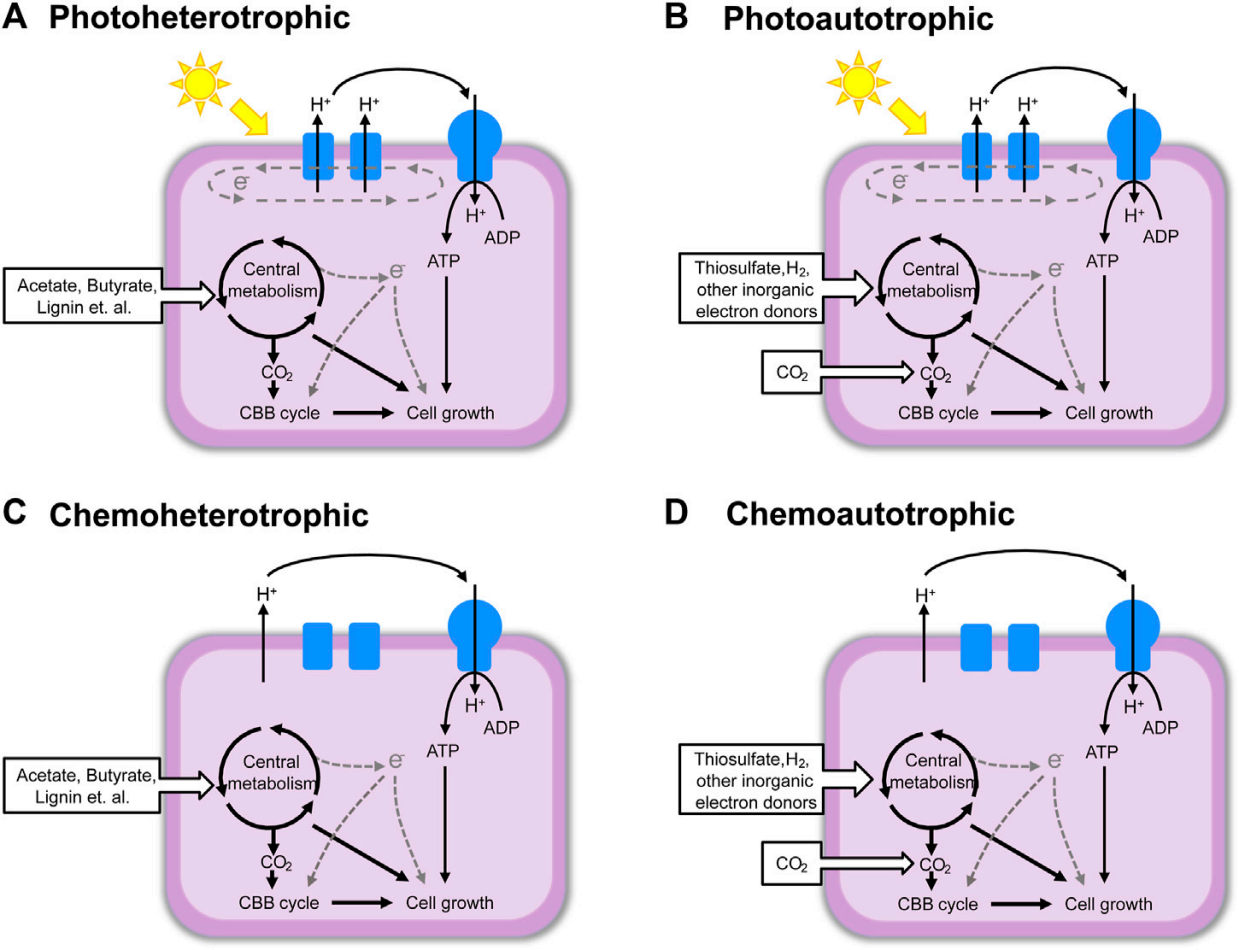
The four types of metabolism of *R. palustris*. **(A)** Photoheterotrophic; **(B)** Photoautotrophic; **(C)** Chemoheterotrophic; **(D)** Chemoautotrophic. This figure was modified according to the figure in reference (Larimer et al., 2003).

*Rhodopseudomonas palustris* belonging to purple nonsulfur bacterium (PNSB) is widely distributed in nature, mainly in anaerobic water environments with sufficient light, such as lakes, soils, swamps, and the sea (Guan et al., 2017). The PNSB constitute a group of versatile organisms in which most exhibit four modes of metabolism: photoautotrophic, photoheterotrophic, chemoheterotrophic and chemoautotrophic, switching from one mode to another depending on conditions available (Figure 1) (Larimer et al., 2003). This metabolic versatility allows *R. palustris* to use light, inorganic, and organic compounds as its carbon and energy sources under anaerobic or aerobic conditions (Madigan and Jung, 2009). Therefore, *R. palustris* is of interest for all sorts of industrial and environmental applications.

Traditionally, *R. palustris* is widely utilized in the areas of aquaculture industry and wastewater treatment. It is rich in biologically active chemicals, including proteins, vitamins, polysaccharides, pantothenic acid and folic acid, which contribute to its function as feed supplement. It can also increase the oxygen content of aquaculture water, stabilize the pH and purify the aquaculture water environment (Peirong and Wei, 2013). The addition of *R. palustris* to aquaculture water can improve the immunity of aquatic organisms and prevent diseases (Zhou et al., 2010). *R. palustris* has biodetoxification and biodegradation capacities toward components in livestock waste and industrial waste, such as lignin, nitride, chlorides and aromatic compounds (Harwood et al., 1998). Analysis of the complete genome sequence of *R. palustris* indicated a large inventory of genes encoding the four distinct ring cleavage pathways for degradation of the aromatic compounds, confirming its outstanding ability of biodegradation (Larimer et al., 2003).

In addition to the above applications, *R. palustris* has great potential to be applied in many other fields. As a microbial cell factory, *R. palustris* has been extensively researched for hydrogen production (McKinlay and Harwood, 2010b) *via* photo-fermentation or *via* complementation of photo-fermentation and dark-fermentation (Patel et al., 2012). Other biofuels production in *R. palustris*, such as methane (Fixen et al., 2016) and butanol (Doud et al., 2017), has also been reported. Production of valuable chemicals in *R. palustris*, such as poly-beta-hydroxybutyrate (PHB) (Wu et al., 2012), polysaccharide (Fritts et al., 2017) and isoprenoid (Xu et al., 2016), indicates the potential of *R. palustris* as a chassis organism for biopolymers and their building blocks production. Moreover, *R. palustris* can transfer electrons to solid electron acceptor such as electrode, enabling its application in microbial fuel cells (MFC) for electricity production (Lai et al., 2017). *R. palustris* also has the ability of electrons uptake from solid materials, leading to its application in the field of microbial electrosynthesis (MES) (Xing et al., 2008; Rengasamy et al., 2018). Similarly, *R. palustris* can absorb electrons from CdS nanoparticles attached to the cell surface, and a CdS-*R. palustris* hybrid system has been constructed for photocatalytic synthesis (Wang et al., 2019).

In this review, the advantages of *R. palustris* as a microbial cell factory were analyzed. Then, the applications of *R. palustris* in biopolymers and their building blocks production were discussed. Traditional application of *R. palustris* in the fields of wastewater treatment, bioremediation, agriculture, aquaculture and livestock breeding were summarized. Application in other fields, including biofuel production, microbial fuel cells, microbial electrosynthesis and photocatalytic synthesis, were also summarized. Finally, the challenges of applications of *R. palustris* are analyzed, and possible solutions are suggested.

## 2 Advantages of *R. palustris* as a Microbial Cell Factory

### 2.1 Utilization of Diverse Carbon Sources, Including CO_2_

*R. palustris* can utilize various carbon sources for growth, including small molecule organic acids, alcohols, inorganic and aromatic compounds, which are the main components in industrial waste (Table 1). *R. palustris* can absorb light as an energy source to produce ATP. Therefore, *R. palustris* has great advantages as a chassis organism in terms of carbon and energy sources.

**TABLE 1:** Carbon sources utilized by *R. palustris*.

Category	Carbon source	References
Short-chain organic acid	Acetic acid, butyric acid, fumaric acid, succinic acid, cyclohexane carboxylic acid, lactic acid, malic acid.	Rey et al. (2007), Adessi et al. (2016), Fixen et al. (2016), Lazaro et al. (2017)
Alcohol compounds	Ethanol, crude glycerol, butanol.	Pott et al. (2014), Liu et al. (2015), Fixen et al. (2016)
Inorganic compounds	Bicarbonate (thiosulfate as electrons donor)	Zheng et al. (2018)
	Syngas (CO, CO_2_, H_2_, etc.)	Pakpour et al. (2014)
Aromatic compound	Coumaric acid, benzoic acid, acetophenic acid, caffeic acid, cinnamic acid, cyclohexanoic acid, ferulic acid, ρ-hydroxybenzoic acid, vanillic acid, syringic acid, etc.	Dutton and Evans (1969), Harwood and Gibson (1988), Harwood et al. (1998), Austin et al. (2015)

First, *R. palustris* CGA009 can use inorganic matters as the carbon source, such as CO_2_, which is fixed by the Calvin Bassham Benson (CBB) cycle to participate in cell growth metabolism using thiosulfate as the electron donor (Huang et al., 2010). Generally, photoautotrophic cyanobacteria have received much attention in the field of photosynthesis of valuable biofuels and chemicals because they can fix CO_2_ through oxygenic photosynthesis with an higher efficiency than plant (Melis, 2009). PNSB, another group of photoautotrophic organisms, have received increasing attention in recent years. PNSB can capture CO_2_ under anaerobic conditions, a photoautotrophic mechanism very different from cyanobacteria (Grattieri, 2020). Furthermore, ribulose 1,5-bisphosphate (RuBP) carboxylase/oxygenase (RubisCO) is responsible for CO_2_ fixation by catalyzing the carboxylation of RuBP *via* the CBB cycle. Usually, the form I RubisCOs are phylogenetically divided into a green type, which is present in cyanobacteria, and a red type (Tabita, 1999; Badger and Bek, 2008; Tabita et al., 2008). Several red-type RubisCOs were demonstrated to have higher CO_2_/O_2_ specificity than green-type RubisCOs (Read and Tabita, 1994; Tabita, 1999). Most PNSB species, including *R. palustris*, contain the red-type form I RubisCO with higher CO_2_/O_2_ specificity (Swingley et al., 2009). Moreover, in *R. palustris*, a proportion of CO_2_ and electrons can be directly catalyzed by remodeled nitrogenases to produce methane (Fixen et al., 2016). In total, except for cyanobacteria, PNSB including *R. palustris* has garnered considerable attention for photosynthetic conversion of CO_2_ into value-added chemicals in recent years.

Second, *R. palustris* can degrade and utilize most short-chain organic acids such as acetate, butyrate, fumarate, succinate and lactate, which are often present in agricultural and industrial wastewater. The metabolism of acetate and butyrate assimilation is initiated by converting to acetyl-CoA, which is mainly assimilated by the glyoxylate shunt, with a few carbon flux entering into the TCA cycle and into pyruvate catalyzed by pyruvate dehydrogenase (McKinlay and Harwood, 2010a; McKinlay and Harwood, 2011). Fumarate and succinate are assimilated directly through the TCA cycle (McKinlay and Harwood, 2011). The assimilation pathway of lactate in *R. palustris* has not been reported; however, a gene (*RPA3503*) encoding D-lactate dehydrogenase and a gene (*RPA1136*) encoding lactate permease were predicated in the genome of *R. palustris* CGA009 (Larimer et al., 2003). Under different organic acid conditions, the growth of *R. palustris* and its productivity are different. Seven different organic acid salts were individually added into the culture medium of methane-producing *R. palustris* as carbon sources, and the highest yield of methane was obtained when fumarate was added (Fixen et al., 2016). *R. palustris* cannot readily use lactate as a sole carbon source, and coutilization with other substrates stimulated its consumption (Govindaraju et al., 2019). When different concentrations (2–10 mmol/L) of lactate were added into the culture medium, different hydrogen yields were obtained (Lazaro et al., 2017). Therefore, when *R. palustris* is utilized as a microbial cell factory, screen of the optimal carbon source is necessary.

Third, *R. palustris* can also use alcohols such as ethanol, crude glycerol and butanol as carbon sources. Crude glycerol is the main byproduct in biodiesel industry (Habe et al., 2009). Due to the rapid development of the biodiesel market, large amounts of crude glycerol are produced and treated as industrial waste (Sabourin-Provost and Hallenbeck, 2009). In *R. palustris*, crude glycerol can be converted to hydrogen at a conversion efficiency nearing 90% (Pott et al., 2013; Pott et al., 2014). Based on environmental and economic considerations, efficient utilization of crude glycerol in *R. palustris* is beneficial for its application as a microbial cell factory.

**FIGURE 2:**
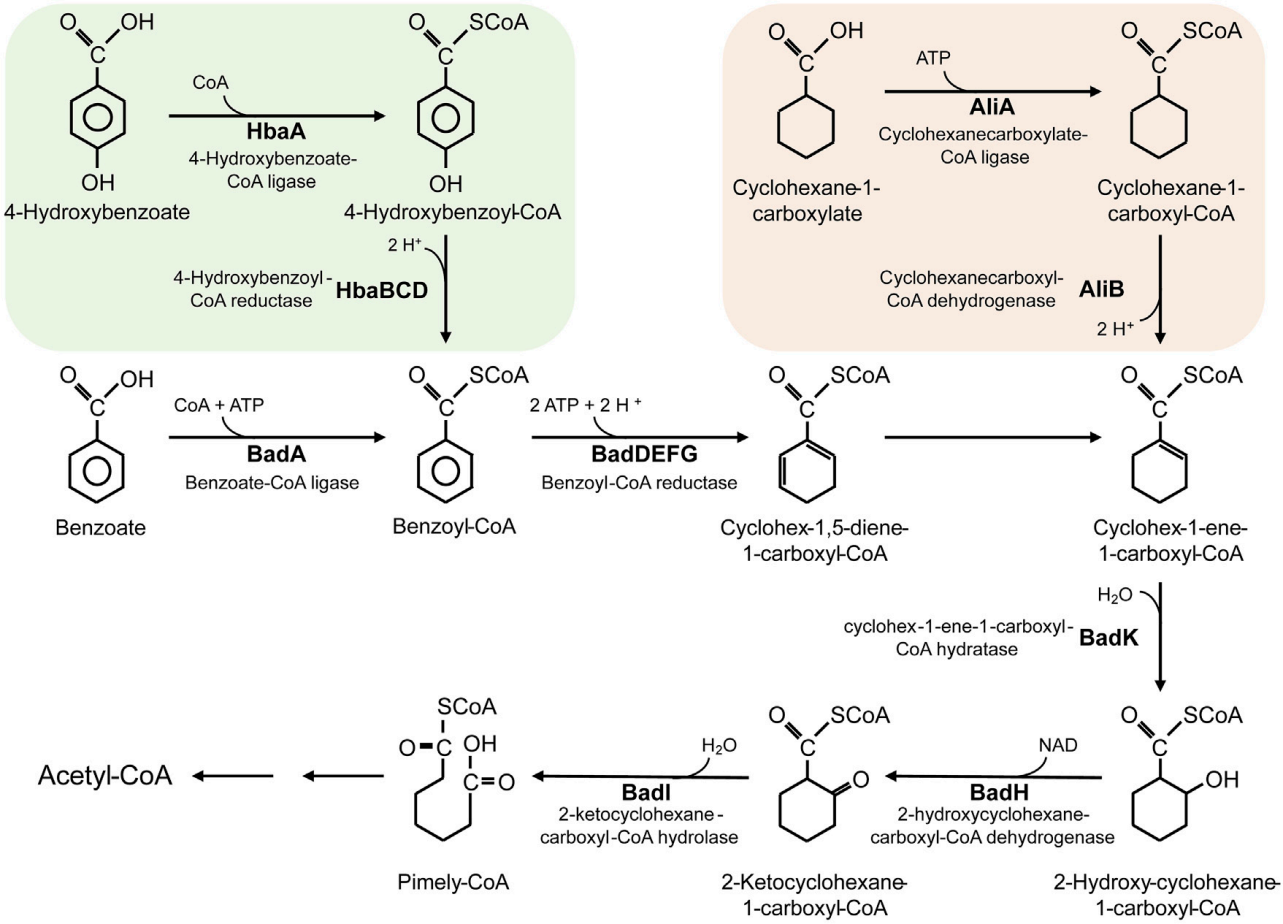
The metabolic pathway for degradation of benzoate, 4-hydroxybenzoate and cyclohexane-1-carboxylate in *R. palustris*.

Last, *R. palustris* can also use multiple aromatic compounds as carbon sources. Utilization of lignocellulosic biomass hydrolysates, an abundant and renewable resource, is the research hotspot in recent years; however, the toxic aromatic compounds in the hydrolysates limit the utilization (Austin et al., 2015). *R. palustris* harbors a benzoate-degrading benzoyl-CoA pathway (Figure 2), through which the aromatic compounds in the hydrolysates are catabolized (Harwood and Gibson, 1988; Egland et al., 1997; Harwood et al., 1998). Conversion of lignocellulosic biomass to valuable chemicals in *R. palustris* is of great value in industrial application.

In summary, due to the diversity of available carbon sources, *R. palustris* has great potential to be utilized as a microbial cell factory for valuable chemicals production, and environmental and economic factors can be considered at the same time.

### 2.2 Exploration of Genetic Engineering Strategies

Development of genetic engineering tools is necessary for manipulation at the genetic level, such as gene knockout and gene overexpression, which can modify the organism as desired. In *R. palustris*, a few gene editing tools have been reported up to date. A plasmid pMG101 (15 kb), which can replicate in PNSB, was obtained from *R. palustris* among 400 strains isolated from a natural environment. Then, based on pMG101, *Escherichia coli*-*R. palustris* shuttle vectors pMG103 (5.68 kb) and pMG105 (5.68 kb) were constructed, which were stably maintained in *R. palustris* (Inui et al., 2000). In *R. palustris* TIE-1, three genes *crtE*, *hpnD* and *dxs* were cloned into pMG103, and squalene production (15.8 mg/g DCW) was obtained (Xu et al., 2016). The pBBR1MCS series, as broad-host-range plasmids, can also be used for vector construction and gene expression in *R. palustris*. The *adhE2* gene was cloned into the pBBR1MCS-2 plasmid, which was then transformed into *R. palustris* CGA009 for *n*-butyrate production (Doud et al., 2017). The overexpression of *fix* gene cluster in *R. palustris* was realized by cloning into the pBBR1MCS-5 plasmid (Huang et al., 2010). Other plasmids in the pBBR1MCS series with different antibiotic selectors, including pBBR1MCS-1, pBBR1MCS-3, and pBBR1MCS-4, can also be used for gene expression in *R. palustris* (Obranic et al., 2013). The pMG103 and pBBR1MCS harbor different origin of replication (*ori*) sources, and co-transformation of pMG103 and pBBR1MCS into *R. palustris* is promising, which is beneficial for overexpression of several genes in the same strain, broadening the application of *R. palustris* as a microbial cell factory.

In addition to gene overexpression, gene mutations mediated by suicide plasmid have also been reported in *R. palustris*. pJQ200SK is a suicide vector that is widely applied in Gram-negative bacteria, featuring the P15A *ori*, a gentamicin selection marker and the gene *sacB* from *Bacillus subtilis*, encoding sucrose-6-fructosyltransferase, which can catalyze the decomposition of sucrose to high-molecular fructan, which has a lethal effect on Gram-negative bacteria (Quandt and Hynes, 1993). The suicide plasmid pJQ200SK was used to insert the mutated nifA* ^*571*^, nifA* ^*574*^, and *nifD* ^*V75AH201Q*^ into the chromosome of *R. palustris* CGA009 to study the function of nitrogenase (Fixen et al., 2016).

To perform more molecular research of *R. palustris* for its further application and characterization, exploring more genetic editing tools is necessary and urgent. In another PNSB, *Rhodobacter sphaeroides*, several other vectors have also been used for gene expression aside from the pBBR1MCS series. The broad-host-range plasmids of the RK2 family, including pRK310, have been widely utilized in *R. sphaeroides* (Keen et al., 1988; Scott et al., 2003; Ryu et al., 2014). The strain harboring pRK310 showed a higher growth rate and higher gene expression levels than the strain harboring pBBR1MCS-2 (Scott et al., 2003). An IPTG-inducible plasmid (pIND4) was also constructed based on plasmid pMG160, and applied for gene expression (Ind et al., 2009; Qiang et al., 2019). In another PNSB, *R. capsulatus*, a set of novel broad-host-range vectors (pRho) with T7 promoters were constructed and further utilized for heterogeneous gene expression for sesquiterpenoids synthesis (Katzke et al., 2010; Troost et al., 2019). Application of these vectors in *R. palustris* is promising, satisfying the requirement of special expression mode in *R. palustris*.

In *R. palustris*, even though the tools for gene overexpression and gene deletion have been reported, the time-consuming genetic editing process limits the widespread application of *R. palustris*. Exploration of novel genetic editing tools is necessary. A SpCas9-sgRNA-based genomic DNA targeting system for *R. sphaeroides* was developed for gene knock-out, knock-in and single nucleotide substitutions, which indicates the development of a similar Cas9-based genome editing tool in *R. palustris* in future research (Mougiakos et al., 2019). In 2020, base editing systems for *R. sphaeroides*, cytosine base editors (CBEs) and adenine base editors (ABEs), all based on CRISPR/Cas9 systems, were also generated (Luo et al., 2020). Development of novel genetic modification tools in *R. palustris* would promote the application of *R. palustris* as a microbial cell factory.

## 3 Applications of *R. palustris*

### 3.1 Application in Biopolymers and Their Building Blocks

Biopolymers are biodegradable polymers that are synthesized from renewable resources by living organisms, which allow them to replace the fossil fuel–based polymers. *R. palustris* is rich in valuable compounds such as PHB, polysaccharide and isoprenoid, building blocks for biopolymers production, making it a promising microbial cell factory (Table 2).

**TABLE 2:** Summary of biopolymers and their building blocks production in *R. palustris*.

Product	Characteristics of main strategies	Yield/titer	Culture conditions	References
3-hydroxybutyrate-co-3-hydroxyvalerate) (PHBV)	Overexpression of *phaP1* from *Cupriavidus necator* H16	0.7 g/L	In PM with 1 mM *p*-coumarate and 10 mM sodium bicarbonate as the carbon courses in sealed 14 ml tubes	Brown et al. (2021)
PHB	An integrated experimental and computational approach to identify novel design strategies	0.41 g/L	In PM with 1 mM *p*-coumarate or coniferyl alcohol supplemented with 10 mM sodium bicarbonate as the carbon sources	Alsiyabi et al. (2021)
PHB	—	0.41 g/L	In PM with 1 mM *p*-coumarate as the carbon source	Brown et al. (2020)
PHB	CdS-*R. palustris* hybrid system.	4% of dry mass	In 50 ml MMN medium with pure CO_2_ gas at the headspace.	Wang et al. (2019)
PHB	Assessment of PHB production under various conditions	5.49 mg/L	Photoelectroautotrophy using N_2_ as the nitrogen source	Ranaivoarisoa et al. (2019)
		6.06 × 10^−14^ mg/cell/h		
PHB	Effect of volatile fatty acids mixtures	16.4 mg/g/day	1,370 mg/L acetic acid, 618 mg/L propionic acid, and 133 mg/L butyric acid	Cardena et al. (2017)
PHB	Assessment of PHB production from agroindustrial residues and energy crops	11.53% TS	In 100 ml photobioreactors with 100 ml olive pomace effluent under anaerobic conditions	Corneli et al. (2016)
PHB	Assessment of PHB production from acetate, propionate, malate, lactate, glucose, and lactose	11.6–17.1% substrate conversion efficiency.	1 g/L acetate	Wu et al. (2012)
Polysaccharide	*R. palustris* contains a functional unipolar polysaccharide biosynthesis gene cluster for polysaccharide production	—	Photoheterotrophic Conditions	Fritts et al. (2017)
Carotenoids	CdS-*R. palustris* hybrid system.	2.5 mg/g dry mass	In 50 ml MMN medium with pure CO_2_ gas at the headspace.	Wang et al. (2019)
Carotenoids	Effect of light sources on growth and carotenoid production	1782 μg/g biomass	In NS medium with 5 g/L sodium succinate as carbon source under LED blue light conditions	Kuo et al. (2012)
Carotenoids	Effect of light intensity and light/dark cycle on carotenoid production	1.94 mg/g biomass	In 0317 medium with volatile fatty acids wastewater under light intensity of 150 μmol-photons/m^2^/s and light/dark cycle of 4/2 (16 h/8 h).	Liu et al. (2019)
Carotenoids	Effect of light intensity and different culturing conditions on carotenoids production and composition	1.5 mg/g biomass (79% lycopene)	In RPP medium with 4 g/L malate and 0.5 g/L NH_4_Cl under hydrogen-production conditions with low light intensity	Muzziotti et al. (2017)
Carotenoids	Effect of hydraulic retention time (HRT) and organic loading rate (OLR) on carotenoid production	3.91 mg/g biomass	Produced from acidic food industry wastewater treated under HRT of 48 h and OLR of 2.51 g/L/d.	Liu et al. (2016)
Squalene	Deletion of the *shc* gene (encoding the squalene hopene cyclase), fusion of two consecutive enzymes (CrtE and HpnD) and overexpression of the *dxs* gene	15.8 mg/g biomass	In medium with 0.2% sodium succinate, 1% glucose, 0.3% peptone, 0.3% yeast extract.	Xu et al. (2016)
Hopanoids	Different growth conditions: chemoheterotrophic, photoheterotrophic and pH shock	36.7 mg/g biomass	Photoheterotrophic growth condition: in anaerobic bicarbonate-buffered freshwater medium with 2 mM sodium acetate	Welander et al. (2009)

#### 3.1.1 Poly-Beta-Hydroxybutyrate Production

Biodegradable polymers, such as polyhydroxyalkanoates (PHAs), have been identified as potential alternatives to traditional plastics, which have been a heavy burden to the environment due to their recalcitrant nature (Kalia et al., 2021). Depending on the carbon atoms per monomer, PHAs can be classified as short-chain-length PHAs (scl-PHAs, C_3_–C_5_) and medium-chain-length PHAs (mcl-PHAs, C_6_–C_14_) (Li et al., 2016). PHAs are accumulated by many bacteria as a carbon- and energy- storage compound, and the most accumulated PHA is PHB, a polymer composed of 3-hydroxybutyrate, belonging to scl-PHAs (Lee, 1996). Although PHB is the most studied PHA in literature, the bioplastic products derived solely from it are typically brittle and stiff, which are undesirable in many applications (McAdam et al., 2020). Nonetheless, the material properties could be improved by adding 3-hydroxyvalerate units into a PHB biopolymer, obtaining copolymer poly(3-hydroxybutyrate-co-3-hydroxyvalerate), also named PHBV (Li et al., 2016). Due to their inherent biodegradability, excellent biocompatibility and non-toxicity, PHAs have been widely applied in the fields of agriculture, aquaculture, and human health sectors (Kalia et al., 2021).

PHB is produced as an energy- and carbon-storage compound by PNSB under special stress conditions (Monroy and Buitrón, 2020). Production of PHB from CO_2_ (carbon source) *via* photoautotrophy in *R. palustris* is considered to be sustainable and environmental-friendly. When the electron donor was a poised electrode (photoelectroautotrophy) or a ferrous iron (photoferroautotrophy), it showed the highest PHB electron yield (7.34%) and specific productivity (8.4 × 10^−14^ mg/cell/h) in *R. palustris* TIE-1 (Ranaivoarisoa et al., 2019). In the CdS-*R. palustris* hybrid system, the CdS nanoparticles cotaded on the surface of *R. palustris* formed by Cd^2+^ bioprecipitation through the cysteine desulfurase can absorb solar energy and release electrons, and the biomass, PHB and carotenoid production were improved by 148%, 147%, and 122%, respectively (Wang et al., 2019). PHB production in *R. palustris* from lignocellulosic biomass which is considered to be the most economic carbon source in the world has received much attention. *R. palustris* can utilize *p*-coumarate, the major lignin breakdown product, to produce PHB (Brown et al., 2020; Alsiyabi et al., 2021). *R. palustris* can produce a copolymer of PHB called PHBV, which has more ideal thermomechanical properties than PHB alone (Li et al., 2016). Phasin has been shown to control the size and number of granules in the cell, affect PHB accumulation, and promote localization of granules, and heterologous expression of phasin in *R. palustris* resulted in a significantly higher PHBV titer (0.7 g/L) from *p*-coumarate (Brown et al., 2021). PHB production from other carbon sources have also been researched, and PHB of different level are obtained with different substrates. Among the different short-chain organic acids utilized, only acetate and propionate can lead to the production of PHB in *R. palustris*, achieving 17.1% and 11.8% substrate conversion efficiency, respectively (Wu et al., 2012). Moreover, mixtures of acetate, propionate and butyrate as substrates had a significant effect on PHB production, 16.4 mg/g/day (Cardena et al., 2017). When acetate was used as the carbon source, under limiting ammonium concentrations, acetate was consumed through the glyoxylate pathway to produce PHB; however, under nitrogen starvation conditions, acetate was consumed through the TCA cycle and PHB production was increased by 30% (McKinlay et al., 2014). Some agroindustrial residues and energy crops were also investigated for PHB formation, and the highest PHB production, 11.53% TS, was obtained in olive pomace effluent (Corneli et al., 2016). In summary, PHB production in *R. palustris* from different carbon sources, like CO_2_, lignocellulosic biomass, short-chain organic acids, agroindustrial residues and energy crops, has been focused. However, in *R. palustris*, hydrogen production competes for the reducing equivalents and metabolites with PHB (Wu et al., 2012), genetic engineering to redirect more metabolic flux from hydrogen to PHB is proposed in future study.

#### 3.1.2 Polysaccharide Production

Polysaccharide biopolymers are composed of mono saccharides linked together by O-glycosidic bonds (Hangasky et al., 2019). Due to their versatile properties like thickening, crosslinking and adsorption, polysaccharide biopolymers have been widely applied in the petroleum industry (Xia et al., 2020). Moreover, based on its properties of low production cost, nontoxicity and biocompatibility, polysaccharides is considered as green biopolymer for *in situ* gel formulation for drug delivery (Chowhan and Giri, 2020). *R. palustris* was reported to produce unipolar polysaccharide for biofilm formation under diverse conditions (Fritts et al., 2017). In the genome of *R. palustris* CGA009, *uppE* (RPA2750) and *uppC* (RPA4833) were identified to be responsible for unipolar polysaccharide production (Fritts et al., 2017). Unipolar polysaccharide production was enhanced in response to three photoheterotrophic conditions, including nutrient limitation, less-preferred nutrients and high salinity; however, cell growth rate was affected (Fritts et al., 2017). Design of the culture conditions of *R. palustris* is of critical importance to balance the cell growth and unipolar polysaccharide production, which affects the overall process economics. Further genetical modification would improve polysaccharide production.

#### 3.1.3 Production of Building Blocks Belonging to Isoprenoids

**FIGURE 3:**
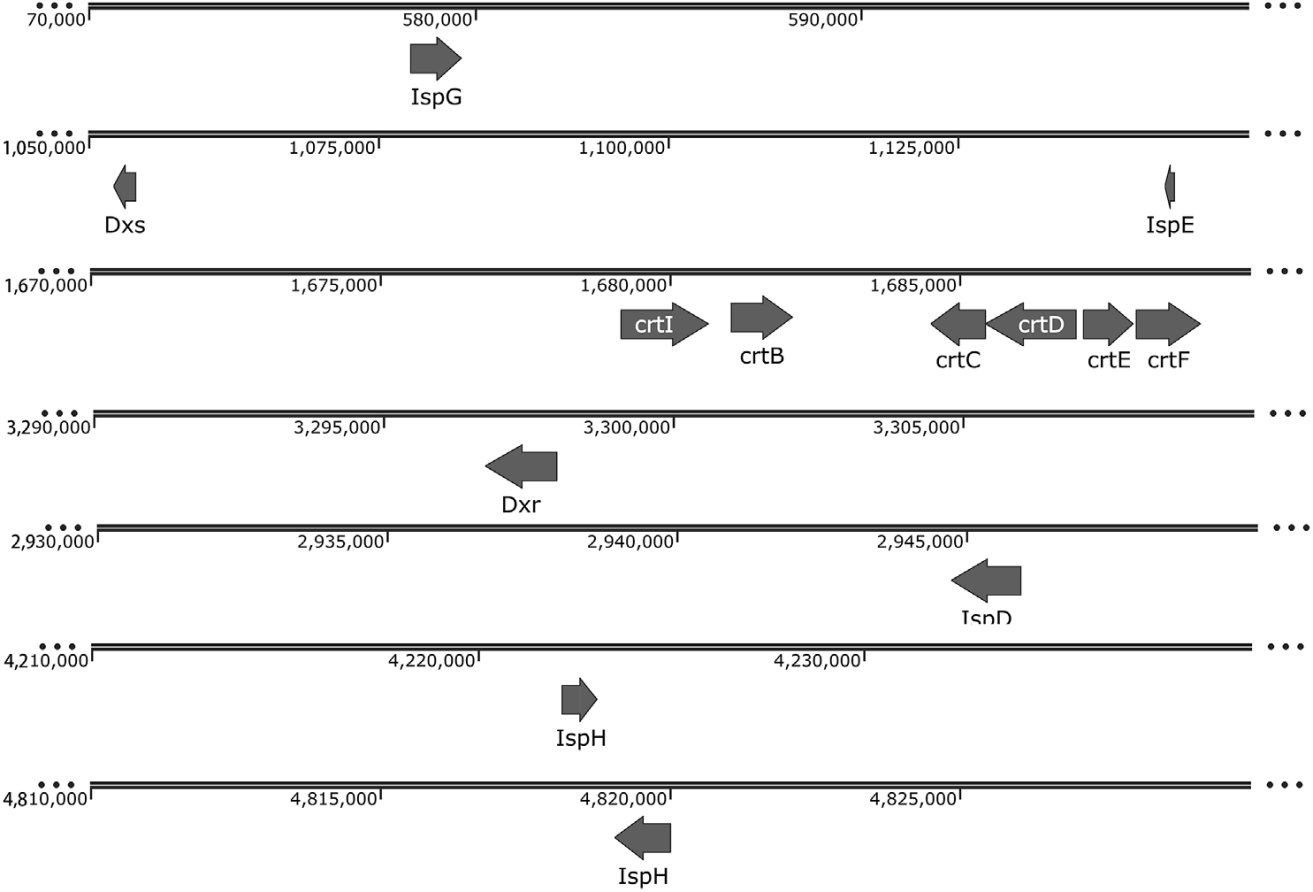
Genome distribution of genes in the carotenoid synthetic pathway in *R. palustris*. Abbreviations: 1-deoxy-D-xylulose-5-phosphate synthase (DXS); 1-deoxy-D-xylulose 5-phosphate reductoisomerase (DXR); 2-C-methyl-D-erythritol 4-phosphate cytidylyltransferase (IspD); 4-diphosphocytidyl-2-C-methyl-D-erythritol kinase (IspE); 4-hydroxy-3-methylbut-2-enyl diphosphate synthase (IspG); 4-hydroxy-3-methylbut-2-enyl diphosphate reductase (IspH); amine oxidase/Phytoene desaturase (crtI), squalene/phytoene synthase (crtB), hydroxyneurosporene synthase (crtC), FAD dependent oxidoreductase (crtD), polyprenyl synthetase (crtE), O-methyltransferase family 2 (crtF).

Among the chemicals belonging to isoprenoids, isoprene and squalene are building blocks for biopolymer production. *R. palustris* is rich in carotenoids, another kind of isoprenoids, which include lycopene, rhodopin, 3,4-didehydro-rhodopin, anhydro-rhodovibrin, rhodovibrin, hydroxy-spirilloxanthin and spirilloxanthin (Chi et al., 2015). The produced carotenoids are connected to the transmembrane proteins through a noncovalent bond, which can stabilize the transmembrane proteins and capture light for the light harvesting complex in the cell membrane (Lang and Hunter, 1994; Takaichi, 2004). The precursors for isoprenoids production, isopentenyl diphosphate (IPP) and dimethylallyl diphosphate (DMAPP), are supplied by the methylerythritol phosphate (MEP) pathway or the mevalonate (MVA) pathway (Li et al., 2019). Genes of the MEP pathway and carotenoid synthetic pathway were identified in the genome of *R. palustris* (Figure 3). The high carotenoids content indicates that *R. palustris* has great potential to be developed as a microbial cell factory for isoprenoids production.

Carotenoids, such as lycopene and β-carotene, have great application value as food additives and in the pharmaceutical industry. Research about carotenoids production by *R. palustris* have been mainly focused on the changes in the culture conditions, such as light sources, light intensity, pH, carbon and nitrogen sources. Among the tested light sources, blue LED showed the highest growth, the highest carotenoid content (1,782 μg/g biomass) and the highest carotenoid productivity (1,800 μg/g/Watt) (Kuo et al., 2012). The light/dark cycle ratio and light intensities also influence the carotenoid yield, and light/dark cycle of 16 h/8 h and light intensity of 150 μmol-photons/m^2^/s were proven to be the best light parameters in *R. palustris* (Liu et al., 2019). Moreover, the composition of the carotenoids are different under different light illumination conditions (Muzziotti et al., 2017). Among the various carotenoids in *R. palustris*, the content of lycopene is the highest, which can reach 79% of the total carotenoids under certain light conditions (Muzziotti et al., 2017). Therefore, lycopene production in *R. palustris* is very promising. Considering the high total carotenoids production in *R. palustris*, further engineering at the gene level would enhance the metabolic flux to a specific carotenoid and isoprenoid.

Squalene production in *R. palustris* has been reported. Due to its antioxidant properties and unique structure, squalene is usually utilized extensively in the pharmaceutical, food, cosmetic and biofuel industries (Kim and Karadeniz, 2012). Squalene derivatives, such as tetramethylsqualene epoxides, botryoxanthins and braunixanthins, could be incorporated to resistant biopolymers by condensation with polyaldehydes (Okada et al., 2000). In *R. palustris* TIE-1, through deletion of the *shc* gene (encoding the squalene hopene cyclase), fusion of two consecutive enzymes (CrtE and HpnD) and overexpression of the *dxs* gene, squalene yield reached 15.8 mg/g (Xu et al., 2016). In another study, the yield of hopanoids, a downstream product of squalene, was more than 36.7 mg/g DCW in wild-type *R. palustris* TIE-1 under certain culture conditions (Welander et al., 2009), which indicates the high potential of further improvement of squalene production.

Isoprene is a platform chemical for natural rubber (*cis*-1,4-polyisoprene) production. Studies on the production of isoprene in *R. palustris* has not been reported; however, our group found that the wild type *R. palustris* can produce isoprene, which was usually produced in plant. Due to its high carotenoids-producing capacity, high isoprene production is promising in *R. palustris*. Isoprene is produced from precursor DMAPP catalyzed by isoprene synthase (Li et al., 2018). Except for the utilization of the native MEP pathway to accumulate DMAPP, heterologous expression of the MVA pathway in *R. palustris* would improve isoprenoid production dramatically. The plasmid harboring the enzymes of the MVA pathway in the vector pBBR1MCS-2 was transformed into another PNSB, *R. sphaeroides*, resulting in an 8-fold increase in amorphadiene production (Orsi et al., 2019). Transformation of the same plasmid into *R. palustris* is expected in future research.

In conclusion, *R. palustris* has great potential to produce biopolymers and their building blocks. *R. palustris* can accumulate biopolymers naturally, such as PHB and polysaccharide, under various conditions. Another beneficial property of *R. palustris* as a microbial cell factory is that industry waste, CO_2_ and lignocellulosic biomass can be converted to valuable biopolymers. The light-harvesting system in the cell membrane of *R. palustris* can capture light as an energy source, contributing to photosynthesis in *R. palustris*. Bioproduction of these valuable chemicals using many traditional chassis organisms, such as *E. coli*, *Saccharomyces cerevisiae*, and *Corynebacterium glutamicum*, has been researched in recent years, and high production has been realized. However, carbon and energy sources of these chassis organisms limit its sustainable and cost-effective application in industry. Therefore, in terms of economy of the fermentation process, *R. palustris* is more suitable as a microbial cell factory for valuable chemicals production. However, bioproduction in *R. palustris* suffers from the low growth rate, and improvement of growth rate is urgently required. Moreover, most research of *R. palustris* has focused on the optimization of culture conditions to improve chemicals accumulation in *R. palustris*; but genetic manipulation of *R. palustris* is seldomly executed due to its complicated genetical engineering process.

### 3.2 Traditional Application

#### 3.2.1 Application in Wastewater Treatment and Bioremediation

**FIGURE 4:**
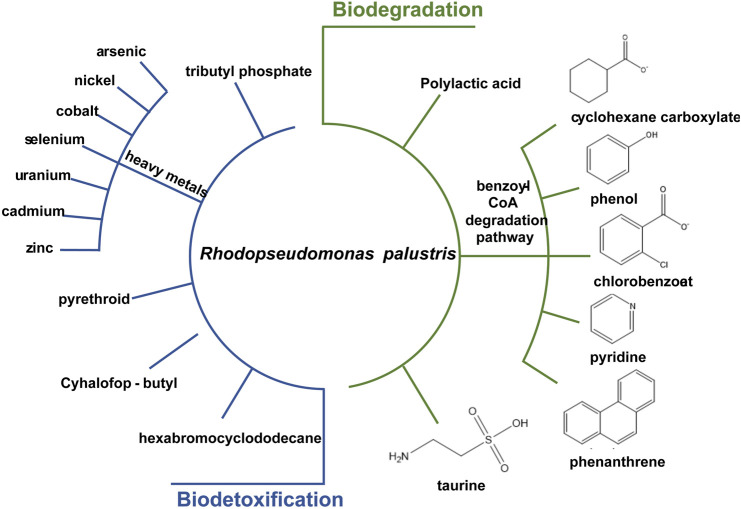
Substrates which can be biodetoxified or biodegraded in *R. palustris*.

*R. palustris* is widely applied in wastewater treatment and bioremediation as a model organism for its biodetoxification and biodegradation properties (Figure 4). Aromatic compounds in the wastewater, such as 3-chlorobenzoate (3-CBA), can be consumed by *R. palustris* as the sole carbon source through the innate benzoate-degrading pathway (Harwood et al., 1998). *R. palustris* WS17 was found to degrade 3-CBA anaerobically in the light; then, *R. palustris* DCP3 was found to degrade not only 3-CBA but also 2-CBA, 4-CBA and 3,5-CBA (Kamal and Wyndham, 1990; van der Woude et al., 1994; Krooneman et al., 1999). The degradation mechanism of 3-CBA in *R. palustris* RCB100 was deciphered, which includes three steps, reductive dechlorination of 3-CBA to 3-chlorobenzoyl coenzyme A, dehalogenation of 3-chlorobenzoyl-CoA to benzoyl-CoA, and degradation of benzoyl-CoA to acetyl-CoA (Egland et al., 2001). *R. palustris* can also grow on cyclohexane carboxylate (CHC), the simplest alicyclic acids, which is degraded to cyclohex-1-enecarboxyl-CoA (CHene-CoA), an intermediate of the benzoate-degrading pathway (Figure 2) (Küver et al., 1995; Hirakawa et al., 2015). Two transcription factors, BadR and BadM, were proven to regulate two operons expression related to the benzoate-degrading pathway (Hirakawa et al., 2015). Additionally, pyridine, a heterocyclic aromatic compound released into the environment as industrial waste, can be degraded by *R. palustris* JA1 as a sole carbon source for growth (Ramana et al., 2002). Another aromatic compound, phenol, is a toxic compound from the chemical industry and human activity, and it can be converted to 4-hydroxyphenylacetate in *R. palustris* PL1 (Noh et al., 2002). *R. palustris* CQV97 was isolated and identified as a microorganism that has the ability to degrade phenanthrene, a polycyclic aromatic compound (Zhao et al., 2011). In addition to aromatic compounds, utilization of taurine, a sulfonate, and degradation of polylactic acid (PLA), have also been reported (Novak et al., 2004; Hajighasemi et al., 2016). The biodegradable PLA is a promising candidate for the replacement of traditional petroleum-based plastics, and depolymerization and recycling utilization of PLA is essential to relieve environment pressure and save resources. The protein RPA1511 from *R. palustris* was identified to have the hydrolytic activity toward PLA (Hajighasemi et al., 2016).

*R. palustris* also has the capability of heavy metal tolerance and assimilation. Heavy metals are released in industrial processes, leading to contaminants in soils and water bodies. *R. palustris* is recognized as a model organism for heavy metal detoxification, especially for arsenic (As), a common soil contaminant. An As-redox transformation system exists in *R. palustris* that can reduce the highly toxic As^5+^ to As^3+^ and oxidize As^3+^ to methylated arsenic (low-toxic) (Batool et al., 2017). The As^5+^ resistance of *R. palustris* is up to 100 mM; and 62.9% of the toxic As^5+^ in the medium can be reduced (Batool et al., 2017). In the genome of *R. palustris*, three *ars* operons and four *arsR* genes related to As metabolism were identified (Zhao et al., 2015). Moreover, cobalt (Co) and nickel (Ni), serious pollutants with the development of industry, can also be assimilated by *R. palustris*. The Co-Ni transporter from *R. palustris* CGA009 was introduced into *Deinococcus radiodurans* R1, resulting in nearly 60% Co removal from the contaminated effluents within 90 min and was introduced into *Nicotiana tabacum* leading to a 5-fold Co acquisition and 2-fold Ni accumulation (Nair et al., 2012; Gogada et al., 2015). In addition, the biodetoxification of other heavy metals, including selenium (Se), uranium (U), cadmium (Cd) and zinc (Zn), have also been reported. After 9 days of cultivation, 99.9% of SeO_3_^2−^ was reduced to red elemental selenium in *R. palustris* strain N (Li et al., 2014). U can be reduced to U(IV)-phosphate or U(IV)-carboxylate compounds or accumulate in the cytoplasm and the cell wall in *R. palustris* (Llorens et al., 2012). Removal of Cd and Zn by 84% and 55%, respectively, were detected in *R. palustris* TN110 (Sakpirom et al., 2017).

In addition to heavy metals, *R. palustris* has high tolerance and degradation effects toward pesticides and herbicides, which are widely applied in agriculture and forestry. The residual pesticides and herbicides in the agro-ecosystem have caused significant environmental and human health concerns in recent years (Katsuda, 1999; Ray and Fry, 2006). Among them, pyrethroid pesticides have been extensively utilized for 30 years. *R. palustris* JSC-3b has the ability to degrade pyrethroid effectively; and the gene *est3385*, encoding a pyrethroid degradation ester, was identified in strain PSB-S (Zhang et al., 2014; Luo et al., 2018). Cyhalofop-butyl herbicides remaining in the soil can also cause serious environmental pollution and risk. The cyhalofop-butyl in soybean processing wastewater can be completely degraded after 5 days by *R. palustris* (Wu et al., 2019). Other toxic compounds detected in various environmental matrixes, such as hexabromocyclododecane and tributyl phosphate, which can cause serious human health problems, can also be degraded by *R. palustris* (Berne et al., 2005; Chang et al., 2020).

Food waste generated from restaurants and schools has caused many management problems in recent years. *R. palustris* CGA009 was reported to remove a wide variety of organic compounds, ammonia, nitrate and starch from food waste, and BOD and COD were reduced by 70% and 33%, respectively (Mekjinda and Ritchie, 2015).

An obvious characteristic of *R. palustris* is the possibility of simultaneous wastewater treatment and valuable chemicals bioproduction. When soybean processing wastewater was adopted as a carbon source, the toxic cyhalofop-butyl in the wastewater was degraded and the production of single cell protein, carotenoid and bacteriochlorophyll were enhanced (Wu et al., 2019). A maximum of 80 ml H_2_/g COD/day hydrogen was concurrently produced with efficient removal of BOD, COD, ammonia, nitrate, starch in the food waste (Mekjinda and Ritchie, 2015).

These outstanding functions make *R. palustris* a potential organism to be applied in wastewater treatment and bioremediation. However, due to the cell washout and unstable biodegradation, these traditional use of suspended *R. palustris* is inefficient. Immobilization microorganism technology, which loads bacteria into a solid carrier to enhance the abundance of bacteria in the wastewater, is attempted in recent years. The immobilized *R. palustris* with alginate, polyvinyl alcohol or agar beads showed 30%∼40% higher ammonia removal rate than that by free *R. palustris* (Zhan and Liu, 2013). *R. palustris* P1 was immobilized to glass pumice with a high adsorption capacity of 4.02 × 10^8^ cells g^−1^, and the maximum NH_4_^+^-N and NO_2_^−^-N removal rates were 134.82 ± 0.67% and 93.68 ± 0.14% higher than those of free *R. palustris* P1, respectively (Xiao et al., 2019). The immobilized technology showed properties like high removal stability, easy to use in continuous reactors, high cell densities, and protecting the bacteria from predation by plankton, contributing to its highly effective aquaculture wastewater treatment.

#### 3.2.2 Applications in Agriculture, Aquaculture and Livestock Breeding

*R. palustris* is traditionally utilized as a biofertilizer in agriculture, and as food additives and water purification microbes in aquaculture and livestock breeding. In agriculture, due to the detoxification and degradation properties of *R. palustris* mentioned above, it can be utilized to clean up the pollutants in the soil and improve the soil quality. An alternative method is to clone the specific genes from *R. palustris* into plants, which process endows the plants with the ability to tolerate and degrade heavy metals and pesticides in the soil. *R. palustris* also has the capability to promote plant growth due to its function of nitrogen fixation (Oda et al., 2005) and the production of two phytohormones, indole-3-acetic acid and ALA (Koh and Song, 2007). Plant *Vigna mungo* treated with *R. palustris* CS2 showed a 17% increase in shoot length and a 21.7% increase in root length (Batool et al., 2017). Additionally, *R. palustris* has the ability to induce resistance against plant disease, such as tobacco mosaic virus (TMV), one of the most destructive plant viruses. The Rhp-PSP protein with inhibitory activities against TMV *in vivo* and *in vitro* was isolated and purified from *R. palustris* JSC-3b (Su et al., 2015). An *R. palustris* GJ22 suspension was sprayed on tobacco, which induced tobacco resistance against TMV and enhanced the immune response under subsequent TMV infection (Su et al., 2017). The growth and germination of the tobacco were also promoted at the same time (Su et al., 2017). These outstanding properties, detoxification and degradation functions, plant growth promoting effects and anti-virus abilities, make *R. palustris* a potential candidate as a biofertilizer in agriculture.

*R. palustris* is also widely applied in aquaculture and livestock breeding in terms of its probiotic properties as feed additives and its wastewater treatment ability. Supplementing the drinking water of broiler chickens with *R. palustris* resulted in the growth promotion and the improvement of meat quality (Xu et al., 2014). The growth performance and immune response of tilapia (*Oreochromis niloticus*) were also improved by using *R. palustris* as a water additive (Zhou et al., 2010). In addition, the probiotic properties of *R. palustris* were further demonstrated in a model animal, rats (Fang et al., 2012). Tissue damage and bacteria translocation were not detected in rats, and no *R. palustris* remained in the feces of the rats 3 days after the termination of intake, indicating the biological safety of *R. palustris* as a probiotic bacterium (Fang et al., 2012). However, the molecular mechanism of its probiotic properties is not very clear. In 2019, an extracellular polysaccharide RPEPS-30 from *R. palustris* was found to enhance the host immune system and improve the growth of beneficial microbes in the gut (Zhang et al., 2019). As we mentioned above, *R. palustris* is suitable for wastewater treatment, which is generated from the aquaculture and livestock breeding industries. *R. palustris* WKU-KDNS3 was isolated from an animal waste lagoon, and its capability to eliminate skatole, a major contributor to the malodor emission from feces, was proven (Sharma et al., 2015). In another study, the isolated *R. palustris* from eutrophicated ponds can remove the odorous organic acids and phosphate swine wastewater (Kim et al., 2004). At present, research about the applications of *R. palustris* in the aquaculture and livestock industries have focused on the isolation of strains and demonstrations of their activity. Further analysis of the molecular basis of its probiotic properties is necessary, and subsequent genetic manipulation of *R. palustris* would improve its probiotic properties.

### 3.3 Application in Other Fields

#### 3.3.1 Application in Biofuel Production

Considering the environmental pollution caused by the consumption of large amounts of nonrenewable fossil fuels, it is urgent to find alternative clean energy sources. Numerous studies have shown that *R. palustris* can produce biofuels, such as hydrogen, methane, ammonia and butanol (Table 3).

**TABLE 3:** Summary of biofuels production in *R. palustris*.

Product	Characteristics of main strategies	Yield/titer	Culture conditions	References
Hydrogen	Screening of mutants that produce hydrogen constitutively, even in the presence of ammonium.	332 μmol/mg protein	In PM medium with 4.5 mM *p*-coumarate by *R. palustris* mutant CGA574	Rey et al. (2007)
Hydrogen	Disruption of the PHB synthesis gene *phbC*	457 ml/L/day	In a 2.5 L bioreactor using organic acid synthetic wastewater	Yang and Lee (2011)
Hydrogen	Influence of light energy and electron availability on CH_4_ production, including providing cells with different substrates, using nongrowing cells, blocking electrons from entering the Calvin cycle, or blocking H_2_ uptake	500 μmol/mg protein	Nongrowing cell suspensions were incubated in light for 10 days in PM with 20 mM acetate and 10 mM NaHCO_3_; *R. palustris* strain nifA* nifD ^*V75AH201Q*^	Zheng and Harwood (2019)
Hydrogen	Evaluation of lighting systems, carbon sources	4.92 mol H_2_/mol substrate	In 30 mM medium with 2 g/L butyrate under incandescent light	Hu et al. (2018)
Hydrogen	Effect of volatile fatty acids mixtures	391 ml/g/day	1.2 g/L acetic acid, 0.2 g/L propionic acid, and 0.05 g/L butyric acid	Cardena et al. (2017)
Hydrogen	—	34 ml/g/h	10 mM glycerol and 5 mM glutamate	Pott et al. (2013)
Hydrogen	Effects of light intensity, the concentrations of crude glycerol and glutamate	6.69 mol/mol glycerol	A light intensity of 175 W/m2, 30 mM glycerol, and 4.5 mM glutamate,	Ghosh et al. (2012)
Hydrogen	Effect of different liming nitrogen regimes	6 mol/mol glycerol	In RCV medium with 10 mM crude glycerol and 2 mM glutamate	Sabourin-Provost and Hallenbeck, (2009)
Hydrogen	Assessment of hydrogen production from agroindustrial residues and energy crops	648.6 mg/L	In 100 ml photobioreactors with 100 ml wheat bran effluent under anaerobic conditions	Corneli et al. (2016)
Hydrogen	Application of immobilized-cell technology	11.2 mmol/m_2_/h 70 ml/h flow rate 0.25 mol/mol glucose	Biofilm formed under 590 nm and 5,000 lx illumination	Liao et al. (2010)
Hydrogen	Application of a cell immobilization technique to a biofilm-based photobioreactor	38.9 ml/L/h 0.2 mol/mol glucose	Illumination condition of 5,000 lux and 590 nm wavelength; glucose concentration is 0.12 M, the optimal pH = 7 and optimal temperature of influent liquid 25°C.	Tian et al. (2010)
Hydrogen	Latex coating immobilization on chromatography paper	0.47 ± 0.04 mmol/m^2^/h	Incubated in the head-space of the Balch tubes	Gosse et al. (2012)
Hydrogen	Effect of osmoprotectants, temperature, humidity and O_2_ on H_2_ production in *R. palustris* coatings	69.1% headspace	*R. palustris* coatings containing glycerol and sucrose after storage for 8–12 weeks at <5% relative humidity; production under argon 10 days post rehydration in PM (NF) medium	Piskorska et al. (2013)
Hydrogen	Blocking of the calvin cycle flux; effect of different substrate	80 mol/mol C consumed	In PM with 10 mM butyrate in front of a 60-W light bulb at 30°C; strain *R. palustris* CGA679	McKinlay and Harwood (2011)
Methane	*nifD* ^*V75A and H201Q*^ encoding a Mo-dependent nitrogenase; calvin cycle through genetic mutation*ΔcbbMLS*; using nongrowing cells; effect of different substrates	800 nmol/mg total protein	Nongrowing cell suspensions were incubated in light for 10 days in PM with 20 mM acetate and 10 mM NaHCO_3_; *R. palustris* strain nifA* nifD ^*V75A H201Q*^	Fixen et al. (2016)
Methane	Influence of light energy and electron availability on CH_4_ production	900 nmol/mg total protein	Nongrowing cell suspensions were incubated in light for 10 days in PM with 10 mM succinate and 10 mM NaHCO_3_; *R. palustris* strain nifA* nifD ^*V75A H201Q*^	Zheng and Harwood (2019)
Methane	V-dependent nitrogenase (*VnfD* ^*V57A H180Q*^); Fe-only nitrogenase	150 nmol/mg total protein (V); 400 nmol/mg total protein (Fe)	Non-growing cell suspensions incubated in light for 10 days in 10 ml NFM medium supplemented with 20 mM acetate and 10 mM NaHCO_3_	Zheng et al. (2018)
Butanol	Overexpression of *adhE2* gene	1.5 mM; 0.03 g/L/day	Cultured in anaerobic butyrate Rhodospirillaceae medium	Doud et al. (2017)

**FIGURE 5:**
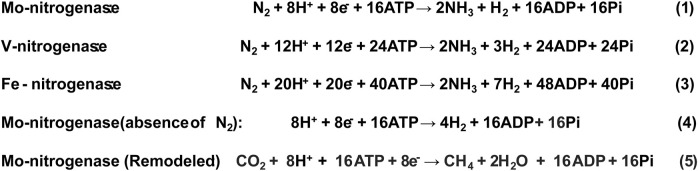
Stoichiometries of production of ammonia, hydrogen and methane through nitrogenase in *R. palustris*. The references were (McKinlay and Harwood, 2010b; Yang et al., 2012; Fixen et al., 2016; Harwood, 2020).

Ammonia is a clean fuel with features like high energy density, no carbon emission, low storage cost, difficult to explode (Bélanger-Chabot et al., 2015; Jiao and Xu, 2019). Ammonia is particularly considered as a strong option for long-haul shipping (Phillips et al., 2010; Zhao et al., 2021). Ammonia is chemically produced from hydrogen and nitrogen by the Haber-Bosch process that is conducted at a high temperature and high pressure in industry (Kandemir et al., 2013). In nature, ammonia can be produced by biological nitrogen fixation catalyzed by nitrogenase (Figure 5, Eqs 1–3), which is highly sensitive to oxygen (Oelze and Klein, 1996; Vitousek et al., 1997). In *R. palustris*, three different nitrogenase gene clusters, *anf*, *vnf* and *nif*, encoding three nitrogenase isozymes, were identified by genomic analysis, and further functional analysis demonstrated their function (Larimer et al., 2003; Oda et al., 2005). Different from algae and cyanobacteria, in which the inhibitory effect of oxygen on nitrogenase limits its wide application, *R. palustris* is an anoxygenic photosynthetic bacteria and the inhibitory effect of oxygen is absent (McKinlay and Harwood, 2010b). Hydrogen and methane production catalyzed by nitrogenase in *R. palustris* have also been reported. Actually, researchers have focused more on hydrogen and methane production than ammonia production by *R. palustris*.

Hydrogen has been recognized as promising alternative due to properties like high conversion efficiency, renewability, clean burning nature and high mass-based energy density (Patel et al., 2012; Hu et al., 2018). Biological hydrogen production has significant potential in the hydrogen economy (Christopher and Dimitrios, 2012). Photo-fermentative hydrogen production by photosynthetic bacteria can be using energy directly from sunlight and reduced organic compounds (Hallenbeck and Ghosh, 2009). In *R. palustris*, hydrogen is produced as an obligate product during the nitrogen reduction process catalyzed by nitrogenase (McKinlay and Harwood, 2010b; Heiniger et al., 2012) (Figure 5, Eqs 1–3), even in the condition of nitrogen deficiency (Figure 5, Eq. 4) (McKinlay and Harwood, 2010b). In this process, electrons are obtained from organic or inorganic compounds and then transported to ferredoxin through the photosynthetic electron transport chain, which is utilized for hydrogen production *via* nitrogenase in combination with generated ATP (McKinlay and Harwood, 2010b). In *R. palustris*, the wide type nitrogenase consumed 75% reductant (ferredoxin) for ammonia production and the hydrogen production is at a low level (Rey et al., 2007). Moreover, the nitrogenase activity is inhibited by ammonia at the transcriptional and posttranslational level and is also inhibited by ADP, which competes with ATP for binding to the enzyme (Dixon and Kahn, 2004; Heiniger et al., 2012; Zheng and Harwood, 2019). Moreover, in *R. palustris*, PHB is synthesized as an energy- and carbon-storage compound, which competes with the metabolites and reducing equivalents for hydrogen production (Vincenzini et al., 1997). Some genetic engineering strategies were explored to improve hydrogen production efficiency, such as improvement of ammonium tolerance of the nitrogenase, elimination of PHB synthesis and disruption of the Calvin cycle. Regulatory gene *nifA* mutants, which lead to high nitrogenase expression even in the presence of ammonium, were screened, and up to five times hydrogen production (332 μmol/mg protein) relative to the wild type was detected (Rey et al., 2007). Disruption of the PHB synthesis gene *phbC* was performed in *R. palustris* WP3-5, and a 1.7-fold increase in hydrogen content (70%) was obtained (Yang and Lee, 2011). Hydrogen production can be improved by 4.6-fold to 145 μmol/mg protein in a *ΔcbbLS ΔcbbM ΔcbbP* mutant strain, in which electrons entering the Calvin cycle were blocked and redirected to hydrogen production (Zheng and Harwood, 2019). An obvious increase of hydrogen production has been obtained after genetic engineering strategies were applied.

Photo-fermentation of hydrogen suffers from the low light conversion efficiency and high energy demand by nitrogenase, leading to the low hydrogen yield. Most research on the improvement of hydrogen production in *R. palustris* has mainly focused on optimization of basic cultivation parameters, including the substrate, light intensity, pH, temperature and immobilization of the cells. Organic acids, such as acetate, propionate, malate and lactate, can be utilized as substrates to produce hydrogen by *R. palustris* WP3-5 (Wu et al., 2012). Four different carbon sources were evaluated for hydrogen production by *R. palustris* DSM127, and the highest hydrogen yield, 1.69, 2.38, 2.57, and 4.92 mol H_2_/mol substrate, were obtained using acetate (3 g/L), malate (2 g/L), lactate (2.5 g/L), and butyrate (2 g/L), respectively (Hu et al., 2018). However, when butyrate (2 g/L) was utilized as the carbon source, hydrogen production started after 400 h, longer than other carbon sources (Hu et al., 2018). Mixtures of different substrates were evaluated for the distribution of hydrogen and PHB production, and mixtures of propionate and acetate showed the highest hydrogen production, 391 ml/g/day (Cardena et al., 2017). The possibility of using various industrial and agricultural wastewater for hydrogen production has been widely investigated. Photofermentation of crude glycerol, a major side product of the biodiesel industry, to hydrogen was also studied in *R. palustris*, and the conversion rate was improved through adjusting the cultivation conditions (Sabourin-Provost and Hallenbeck, 2009; Ghosh et al., 2012; Pott et al., 2013). Agroindustrial residues and energy crops were also investigated for hydrogen production in *R. palustris*. For instance, a *R. palustris* CGA676 mutant cultured with wheat bran and maize effluent produced 648.6 and 320.3 ml/L hydrogen, respectively (Corneli et al., 2016). Light systems are also essential for hydrogen production. Two light systems, the fluorescent and incandescent, were investigated for cell growth and hydrogen production, and the incandescent system was proven to be more effective (Hu et al., 2018). Immobilization of *R. palustris* through biofilm formation and latex coatings formation can concentrate its biological reactivity and stabilize the microbes, which is beneficial for hydrogen production and light conversion efficiency (Liao et al., 2010; Tian et al., 2010). *R. palustris* CQK 01 was immobilized on the surface of baked glass beads to form a biofilm, and the bioreactor showed high hydrogen production, 38.9 ml/L/h and 0.2 mol/mol glucose (Tian et al., 2010). Latex coatings of *R. palustris* CGA009 were constructed in both dry and wet conditions, and high-level hydrogen production was detected (Gosse et al., 2012; Piskorska et al., 2013). In summary, hydrogen production by *R. palustris* has been widely studied, and abundant genetic engineering strategies and optimization of culture conditions have been performed.

Moreover, the intracellular redox balance can also influence hydrogen production in *R. palustris*. During photoheterotrophic conditions, *R. palustris* will produce a large amount of reducing equivalents, a part of which are consumed by nitrogenase for hydrogen production for intracellular redox balance (McKinlay and Harwood, 2011). However, the native Calvin cycle can also absorb a large section of the reducing equivalents (McKinlay and Harwood, 2010a; McKinlay and Harwood, 2011). The CbbRRS system in the Calvin cycle can respond to the redox signal and regulate the carbon flux (Romagnoli and Tabita, 2007; Laguna et al., 2010). Therefore, the Calvin cycle competes for reducing equivalents with hydrogen production. When the Calvin cycle was blocked in *R. palustris*, the hydrogen produced was enhanced on all substrates (McKinlay and Harwood, 2011). Hence, for hydrogen production, attention should be paid to the oxidation state of substrates and the Calvin cycle flux to avoid redox imbalance.

The efficiency of hydrogen production can be further improved *via* complementation of photo-fermentation and dark-fermentation. In this sequential two-stage dark- and photo-fermentation process, dark hydrogen production was conducted using acidogenic bacteria like *Clostridium butyricum* (Su et al., 2009a), *C. pasteurianum* (Chen et al., 2008), *Caldicellulosiruptor saccharolyticus* (Özgür et al., 2010), and *E. coli* (Sangani et al., 2019), and photo hydrogen production was conducted using photosynthetic bacteria, like *R. palustris*. A sequential dark- and photo-fermentation system including *C. pasteurianum* and *R. palustris* has been constructed for hydrogen production using sucrose as a feedstock (Chen et al., 2008). The overall hydrogen yield increased from the maximum of 3.8 mol H_2_/mol sucrose in dark fermentation to 10.02 mol H_2_/mol sucrose by sequential dark and photofermentation (Chen et al., 2008). In the sequential dark- and photo-fermentation system, photo-fermentation can utilize the resulting effluent from dark fermentation, mainly consisting of butyric and acetic acid. It is reported that the sequential dark- and photo-fermentation system could achieve a theoretically maximum hydrogen yield (Su et al., 2009b); therefore, is has been considered as an efficient system to increase hydrogen production yield.

Methane is a commonly used energy source, and nearly half of hydrogen production in industry is from methane. A purified Mo-dependent nitrogenase from *Azotobacter vinelandii* with two mutations, V70A and H195Q, in *NifD* near the FeMo cofactor, was proven to be capable of reducing carbon dioxide to methane *in vitro* (Yang et al., 2012). Then, homologous mutations, V75A and H201Q, in *NifD* from *R. palustris*, were produced and methane production from carbon dioxide was also detected *in vivo* (Figure 5, Eq. 5) (Fixen et al., 2016; Zheng and Harwood, 2019). Further optimization indicated that utilization of nongrowing cells which are not carrying out the competing biosynthesis can enhance the yield of methane by 9-fold to 900 nmol/mg total protein using fumarate as the substrate (Zheng and Harwood, 2019). The analogous mutation of the V-dependent nitrogenase (*VnfD* ^V57AH180Q^) can also catalyze the synthesis of methane in *R. palustris* (Zheng et al., 2018). The wild type of another Fe-only nitrogenase in *R. palustris* also has the ability to synthesize methane, ammonia and hydrogen simultaneously, but the proportion of methane is relatively low (Zheng et al., 2018). With in-depth study of the mechanisms of nitrogenase, improving the ability of nitrogenase to synthesize hydrogen and methane through genetic engineering and synthetic biology methods are the direction of further research.

In addition, the production of butanol by *R. palustris* has also been reported. Compared to ethanol, butanol is an attractive renewable biofuel with a higher energy content, lower volatility, higher viscosity and higher intersolubility with traditional fuels (Jin et al., 2011). The *adhE2* gene from *Clostridium acetobutylicum* ATCC 824 encodes alcohol-aldehyde dehydrogenase, which catalyzes the synthesis of *n*-butanol from *n*-butyrate, was expressed in *R. palustris* (Doud et al., 2017). A selectivity (butanol production from butyrate) up to 40%, close to the theoretical maximum selectivity 45%, was achieved in the engineered *R. palustris* (Doud et al., 2017). However, a very low *n*-butanol production rate, 0.03 g L^−1^ d^−1^, was obtained as a result of the slow growth of *R. palustris*, and the redox flux is speculated to be the limiting factor (Doud et al., 2017). Further engineering to improve its growth rate and metabolic rate is proposed to improve butanol and other biofuels production.

Biofuel production, mostly hydrogen, in *R. palustris* has been widely researched in the fields of diverse substrates and genetical modification to improve the metabolic flux. However, the low production yield still limits its application in industry. Combination of biofuel production with industrial and agricultural wastewater treatment or biodegradation might be a promising strategy to reduce cost.

#### 3.3.2 Application in Microbial Fuel Cells, Microbial Electrosynthesis and Photocatalytic Synthesis

**FIGURE 6:**
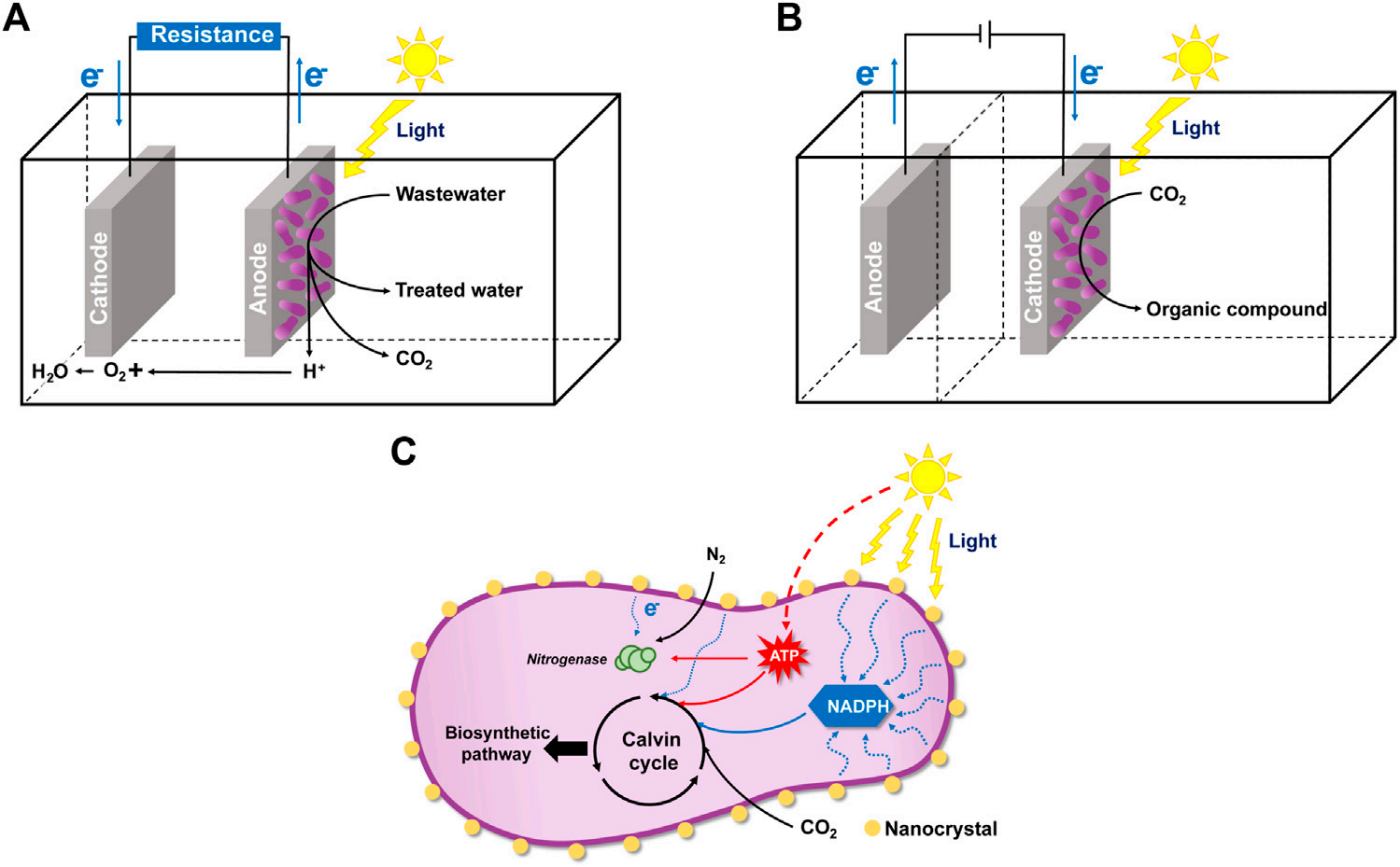
Illustration of the microbial fuel cell (MFC) and microbial electrosynthesis (MES) of *R. palustris* and CdS-*R. palustris* hybrid system. **(A)** In the MFC system, *R. palustris* in the anode can oxidize organic substrates in the wastewater and release electrons and protons. Electricity current is generated, and the protons are transferred to the cathode and react with oxygen to generate water. **(B)** In the MES system, the electrons from the cathode is absorbed by *R. palustris* for organic chemicals production from inorganic carbon dioxide and light energy. **(C)** In the CdS-*R. palustris* hybrid system, *R. palustris* can absorb electrons from the CdS nanoparticles (NPs) coated on its cell surface.

In recent years, some novel application fields were reported for *R. palustris*, utilizing its electron exchange ability with solid-phase materials, such as MCF, MES and photocatalytic synthesis (Figure 6). Construction of MFC has attracted much attention in recent years due to its electricity generation ability. In the MFC system, microbes can oxidize organic substrates in the anode and release electrons and protons, which are transferred to the cathode and react with oxygen to generate water, while generating electricity at the same time (Figure 6A) (Slate et al., 2019). Photo-microbial fuel cells (PMFC), which can convert light energy to electricity due to the photosynthetic activity of the photosynthetic microorganisms, are also being developed. *R. palustris* has been utilized as a model organism in PMFC. The power density of *R. palustris* DX-1 achieved 2,720 mW/m^2^ under light with acetate as the electron donor (Xing et al., 2008). Carbon sources from wastewater and contaminated sites can also be utilized as electron donors in the PMFC system. *R. palustris* RP2 can produce a current (power density 720 μW/m^2^) using petroleum hydrocarbons from oil contaminated sites as the energy source (Venkidusamy and Megharaj, 2016). In another study, *R. palustris* G11 could accumulate polyphosphate and PHB when a sufficient carbon source was provided, and the stored polyphosphate and PHB were further used for electricity production in a carbon source-insufficient condition (Lai et al., 2017). Power production under carbon source-insufficient conditions indicates the simultaneous application of PMFC for energy production and wastewater treatment, in which the carbon source is not enriched. Recently, a novel hybrid hydrogen-photosynthetic microbial fuel cell was constructed, which can produce hydrogen and a maximum power density of 2.39 mW/m^2^ in *R. palustris* ATCC 17007 at the same time (Pankan et al., 2020). The performance of PMFC may be affected by the electrode material, reactor, membrane, substrate and operating conditions (Aghababaie et al., 2015). Application of PMFC is mostly limited by its low power generation and expensive electrode materials, and research should be focused on improving the rate of electron transfer to/from the electrode and the exploration of advanced materials as the electrode.

Microorganisms applied in MCF can release electrons to the solid-phase material and generate electricity. However, microorganisms can also absorb electrons from the cathode for MES, a bioelectrochemical process (Rabaey et al., 2011). In an MES system, desired chemicals are produced from an inorganic substrate and the absorbed electrons at the cathode-microbe interface (Figure 6B) (Karthikeyan et al., 2019). Photoautotrophic iron-oxidizing *R. palustris* TIE-1 can accept electrons from a solid-phase electrode, with carbon dioxide as the sole carbon source and electron acceptor (Bose et al., 2014). In *R. palustris* TIE-1, the *pio* operon is responsible for electron uptake from the electrode, and electron uptake stimulates *ruBisCo* form I expression, indicating the enhancement of carbon fixation during MES (Jiao and Newman, 2007; Bose et al., 2014). The major obstacle for using *R. palustris* TIE-1 for MES is the low electron uptake efficiency from cathode, which is reflected by the low maximum current density values (Ranaivoarisoa et al., 2019). To further enhance the electron uptake ability by *R. palustris* TIE-1, several iron-based redox mediators coated cathodes were tested and the immobilized Prussian blue (PB)-coated cathode was proven to be the best (Rengasamy et al., 2018). In another study, to enhance the electron uptake rates of *R. palustris* TIE-1, a photobioelectrochemical system with two uncoupled reactors, an electrochemical system and a photobioreactor, was developed, which had an electron uptake rate 56-fold higher than the single system (Doud and Angenent, 2014). In summary, the application of *R. palustris* in MES has been reported in recent years, but electron transfer efficiency is still the key factor limiting its efficiency. Actually, even after a decade of development, using MES for chemical synthesis cannot compete with the traditional fossil-fuel-derived production (Prevoteau et al., 2020). However, MES is still promising for chemical production from carbon dioxide, and the performance of MES can be improved.

*R. palustris* can also absorb electrons from the nanoparticles (NPs) coated on its cell surface. An inorganic-biological hybrid system, a CdS-*R. palustris* hybrid system, was constructed, in which CdS NPs coated on the cell surface can absorb solar energy, release electrons, then participate in various biosynthetic pathways (Figure 6C) (Wang et al., 2019). In this semiartificial photosynthetic platform, solar energy is directly utilized for the chemical production. CdS NPs on the surface of *R. palustris* are formed by Cd^2+^ bioprecipitation through the cysteine desulfurase (Bai et al., 2009). In the CdS-*R. palustris* hybrid system, carbon dioxide reduction was enhanced and the production of solid biomass, carotenoids and PHB were increased to 148%, 122%, and 147%, respectively (Wang et al., 2019). The synthesized CdS NPs can also locate to the cytoplasm, absorb solar energy, and enhance the nitrogen fixation catalyzed by nitrogenase (Sakpirom et al., 2019). Research about the nanoparticles-photosynthetic hybrid system is still in the initial stage. Further research on different nanomaterials and the genetic modification of *R. palustris* are expected to improve its catalytic efficiency.

## 4 Challenges and Possible Solutions

*R. palustris* has great potential for application in many fields: valuable chemicals production as a chassis organism; wastewater treatment and bioremediation for its biodetoxification and biodegradation properties; agriculture, aquaculture and livestock breeding as food additives; MCF, MES and photocatalytic synthesis for its electron exchange ability with solid materials. However, some properties of *R. palustris* limit its wide application. First, even though the versatile *R. palustris* can use a variety of carbon sources, its metabolic efficiency and growth rate are very low. Random mutation and screening are expected to improve its metabolic efficiency. Second, to our knowledge, except for a few gene expression vectors (pMG103 and pBBR1MCS series) and a gene knockout vector (pJQ200SK), no other genetic manipulation tools available in *R. palustris* have been reported. Exploring more genetic tools for *R. palustris* is necessary. Genetic manipulation tools, such as pRK404 and pIND4, which are available in other PNSBs, may be suitable for *R. palustris*. In addition, the implementation of some novel gene manipulation technologies, such as CRISPR-Cas and antisense RNA, in *R. palustris* is promising. Third, most of the research about *R. palustris* is at the physiological and biochemical levels, and genetic manipulation to enhance its efficiency is rarely reported. For example, for hydrogen production, most of the research has focused on the optimization of culture conditions to improve its production, and only limited research about the genetic manipulation of *R. palustris* has been reported. However, genetic manipulation could improve the properties of *R. palustris* significantly. Development of genetic engineering tools would facilitate genetic manipulation of *R. palustris* to improve its performance.
